# The paracrine effects of tumor cells on endothelial cells and macrophages in brain metastases with different primary sites

**DOI:** 10.3389/fimmu.2026.1872801

**Published:** 2026-08-26

**Authors:** Tamer A. Kaya, Klaus-Peter Stein, Belal Neyazi, Ali Rashidi, Ulf D. Kahlert, Christian Mawrin, I. Erol Sandalcioglu, Claudia A. Dumitru

**Affiliations:** 1 Department of Neurosurgery, Otto-von-Guericke University, Magdeburg, Germany; 2 Molecular and Experimental Surgery, University Clinic for General-, Visceral-, Vascular- and Transplantation Surgery, Otto-von-Guericke University, Magdeburg, Germany; 3 Research Campus STIMULATE, Otto-von-Guericke University, Magdeburg, Germany; 4 Department of Neuropathology, Otto-von-Guericke University, Magdeburg, Germany

**Keywords:** angiogenesis, brain metastases, macrophages, T-cells, tumor progression

## Abstract

**Background:**

Brain metastases (BrMs) represent the most common intracranial tumors, which occur at a significantly higher incidence than primary brain neoplasms. Although some studies addressed the modulation of endothelial cells (ECs) and macrophages in lung and breast BrMs, it is still unclear how these cells are modulated in other BrM types.

**Methods:**

Tumor cells (TCs) were isolated from human melanoma, lung and gastrointestinal (GI) BrM tissues, using individual patient isolates (n=2 per tissue origin) as the primary unit of inference. The paracrine effects of BrM-TCs on ECs and macrophages were examined in *ex vivo* models using BrM-TC-derived supernatants, HUVEC ECs, and peripheral blood monocytes isolated from healthy donors. The functional implications of the macrophages stimulated with BrM-TCs were investigated on autologous T-cells, focusing on T-cell proliferation.

**Results:**

None of the BrM-TCs had a direct effect on the ECs regarding MMP release, migration, proliferation and tubulogenesis. Interestingly, lung and GI but not melanoma BrM-TCs polarized macrophages into an M2-like phenotype, characterized by enhanced MMP9 release, increased Arg1 and CD206 expression, as well as elevated IL-6, IL-8, IL-10, and VEGF levels. Moreover, macrophages polarized by lung and GI but not melanoma BrM-TCs inhibited the proliferation of CD8 and CD4 T-cells.

**Conclusion:**

These findings indicate that lung and GI but not melanoma BrM-TCs polarize macrophages towards a tumor-promoting, M2-like phenotype. Although the direct effects of BrM-TCs on ECs remain to be further elucidated, our results highlight the need for further comparative studies on BrMs, with particular focus on their histological origins.

## Introduction

Brain metastases (BrMs) are the most common malignant tumors of the central nervous system, that occur at a rate approximately ten times higher than primary brain tumors (1). One in every five cancer patients is estimated to develop BrMs during the course of their disease (2). Lung cancer, particularly non-small-cell lung cancer (NSCLC), is the leading source of BrMs, followed by breast cancer and cutaneous melanoma (3). BrMs from gastrointestinal (GI) tumors, which consist mostly of colorectal carcinoma, are relatively rare. However, they are highly aggressive, and are associated with poor clinical outcomes and reduced overall survival of the respective patients (4).

The tumor microenvironment (TME) consists of different cell types that dynamically modulate disease progression. These include -apart from the tumor cells (TCs) themselves- fibroblasts, pericytes, endothelial cells (ECs), as well as tissue-resident and -infiltrating immune cells. It is well known that the interactions between TCs and ECs play an important role in the pathophysiology of brain tumors, including BrMs, since they result in an aberrant angiogenic activity and tumor progression, respectively. The functional changes induced by TCs in ECs include modulation of hypoxic responses, shifts in EC energy metabolism and proliferation, as well as up-regulation of pro-angiogenic factors alongside down-regulation of anti-angiogenic factors (reviewed in (5, 6)). In addition, TCs interact with ECs either directly, via formation of gap junctions, or via modulation of intermediate cell types, such as pericytes or immune cells. These steps are further associated with extracellular matrix remodeling by ECs and with the formation of new blood vessels (reviewed in (6, 7)). However, the differential modulation of the angiogenic cascade depending on the primary site of the BrMs is still poorly characterized at present.

Previous studies on brain tumors, including BrMs, revealed that the TME is infiltrated by monocyte-derived macrophages, which polarize into tumor-associated macrophages (TAMs) (8–11). This infiltration is driven by TME-associated factors, and the inhibition of these factors could prolong survival in murine glioma models (12, 13). Compared with non-tumor macrophages, TAMs upregulate pathways related to inflammatory signaling, cytokine production, cell migration, cell cycle regulation, and extracellular matrix organization (as recently reviewed in (14–17)).

TAMs are generally categorized into anti-tumor (M1) and pro-tumor (M2) phenotypes, which differ in surface marker expression, cytokine secretion, and functional properties (for recent reviews see (14, 18, 19)). However, advances in OMICS technologies revealed that this dichotomous M1/M2 classification oversimplifies the TAM complexity, which instead exists as a spectrum of diverse functional subpopulations (20, 21). For instance, IL-6, which is classically assigned to the M1 phenotype, can also be expressed and/or released by M2 TAMs (22, 23). Similarly, Vogelpoel et al. reported that CD163-positive M2 macrophages selectively amplified the production of pro-inflammatory cytokines IL-6, IL-1β and TNF-α, which are typically associated with M1 phenotype (24). Moreover, recent advances in spatial genomics demonstrated location-specific modulation of TAMs within the brain TME, and enabled the identification of distinct subsets (25, 26). These studies underline the complexity of the TAM phenotypes, as well as the limitations of the binary M1/M2 classification.

TAMs were reported to suppress anti-tumor immunity through multiple mechanisms in brain tumors; however, these findings were predominantly limited to glioblastoma. These mechanisms include the expression of PD-L1 (27, 28), the release of IL-10 and VEGF (29, 30), as well as the upregulation of Arg1 in TAMs (20, 31). Interestingly, Zhu et al. showed that M2-like TAMs suppressed the activation of CD8 T-cells through the secretion of IL-10 and TGF-β in breast cancer BrM (32). Alvarez-Prado et al. compared lung and breast BrMs and reported that TP53^mut^ lung BrMs were enriched in immune-suppresive TAMs compared to KRAS^mut^ or breast cancer BrM (33). Furthermore, Guldner et al. reported that M2-like TAMs recruited T-cells through the upregulation of CXCL10, while simultaneously inducing their exhaustion in murine models of BrM (34).

These findings provide strong evidence that ECs and TAMs contribute to the progression of BrMs. However, as most previous studies focused on lung and breast cancer BrMs, the functions of these cells in BrMs of other origins remains unclear. In this study, we aimed to evaluate the impact of TCs isolated from melanoma, lung and GI cancer BrMs on 1) the angiogenic cascade, including basal membrane degradation, migration, proliferation, and tubulogenesis of ECs, 2) macrophage polarization and TAM phenotype and 3) functional implications of these TAM states on T-cell proliferation.

## Materials and methods

### Material

Details on all material used in this study are listed in supplementary **Supplementary Table 1**.

### Cells and conditioned supernatants

Brain metastasis tumor cells (BrM-TCs) were isolated from tumor tissues of six patients, who had been treated in our department between 2022 and 2023. Monocytes and T-cells were obtained from the peripheral blood of healthy adult volunteers within our institution. All participants provided informed written consent. The study was approved by the ethics committee of the Otto-von-Guericke University (NeuroCAM study 145/19). HR55 and JU61 TCs were derived from melanoma BrM, RA66 and BA69 TCs from lung BrM, and BH64 and BB61 TCs from GI BrM. RA66 and BB61 TCs were isolated from patients that had received chemotherapy prior to the BrM resection, while the donor of BH64 had received both chemo- and immunotherapy. The other three patients did not receive any systemic therapy prior to the BrM resection. Detailed information on further patient characteristics, isolation and characterization of the BrM-TCs, as well as the preparation of conditioned supernatants (SN) is provided in our previous study (35).

Human umbilical vein endothelial cells (HUVEC) were used as an EC model in this study. The cells were kindly provided by the Department of Cardiology and Angiology, Otto-von-Guericke University Magdeburg, but are also commercially available.

### Gelatine zymography

To determine the levels of gelatinases (MMP2 and MMP9), HUVEC were stimulated with BrM-TC SNs for 3 days, washed with PBS, and then allowed to release gelatinases in regular cell culture medium for another 3 days, at a density of 1x10^5^ cells/mL. The samples were subsequently mixed with zymogram sample buffer at a final concentration of 80 mM Tris pH 6.8, 1% SDS, 4% glycerol and 0.006% bromophenol blue. The proteins were separated by SDS-PAGE containing 0.2% gelatin 180 Bloom, and were then renaturated in 2.5% Triton-X-100 for 1 h at room temperature. The enzymatic reaction was carried out in a buffer containing 50 mM Tris pH 7.5, 200 mM NaCl2 and 1% Triton-X-100 overnight at 37 °C. The gels were then stained with a solution containing 0.5% Brilliant Blue G250, 30% methanol and 10% acetic acid for 1 h at room temperature, followed by three de-staining steps with 10% acetic acid and 30% methanol until the digested bands became visible. The bands were analyzed with the ImageJ 1.48v software. These studies included three independent experiment/biological replicates, each with one technical replicate.

The same procedure was applied for macrophages, except that macrophages were stimulated with BrM-TC SNs for 48 h, after which the BrM-TC SNs were removed. The cells were then allowed to release gelatinases in regular cell culture medium for another 24 h, at a density of 1x10^6^ cells/mL. These studies were performed with macrophages from four independent blood donors, each with one technical replicate.

### Migration assay

Migration of HUVEC was measured using the Oris™ system. Initially, 96-well plates were coated with a matrix containing 1 mg/mL collagen I for 30 min at 37 °C, and the cells were seeded in the presence of a stopper to create a cell-free “gap” in the middle of the wells. After the cells adhered, the stoppers were removed, and the cells were subsequently allowed to migrate in the cell-free area for 24 h. The degree of “gap closure” (cell-free area at 0 h vs 24 h) was quantified using the ImageJ 1.48v software. These studies included five independent experiments/biological replicates, each with two technical replicates.

### Proliferation assay

The proliferation of HUVEC was measured using the MTT assay. Initially, the cells were seeded in 96-well plates at a density of 4000 cells/well. At day 4, cell culture medium containing 10% Thiazol Blue (3-(4,5-demithylthiazol-2-yl)-2,5-diphenyltetrazolium bromide) was added for 4 h at 37 °C to allow formation of formazan crystals. Subsequently, the cells were lysed with isopropanol containing 0.33% HCl, and the OD values were measured at 540/690 nm on a TECAN plate reader. These studies included five independent experiments/biological replicates, each with 8 technical replicates.

### Tube formation

The tubulogenesis of HUVEC was evaluated using tube formation assays. Briefly, 96-well plates were coated with 50 µL undiluted Cultrex Basement Membrane Extract for 30 min at 37 °C. The cells were then seeded at a density of 15x10^3^ cells/well, followed by an incubation step of 5 h at 37 °C. The number and length of segments and branches were quantified using the angiogenesis macro of the ImageJ 1.48v software. These studies included three independent experiments/biological replicates, each with 2 technical replicates.

### Monocyte isolation and differentiation into macrophages

Peripheral blood from healthy donors was diluted 1:1 with PBS and subjected to density gradient centrifugation using Pancoll. The mononuclear cell fraction was transferred into a new tube and washed 3 times with PBS. Monocytes were subsequently isolated from mononuclear cell fraction using the Pan Monocyte Isolation Kit, according to manufacturer´s instructions. Isolated monocytes were then incubated in regular cell culture medium supplemented with 50 ng/mL M-CSF for 6 days at 37 °C.

### Analysis of M1/M2 marker expression

Macrophages were seeded in Nunc UpCell 6-well plates and were stimulated with BrM-TC SNs for 48 h, after which the BrM-TC SNs were removed. The cells were then incubated in standard cell culture medium for another 24 h at a density of 1x10^6^ cells/mL. The macrophages were detached from the plates using Macrophage Detachment Solution, according to manufacturer’s recommendations. The effect of BrM-TC SNs on M1/M2 marker expression was assessed by flow cytometric analysis using the following antibodies: CD86-APC-Vio770, CD163-PE, CD206-VioBlue, Arg1-APC, as well as iNOS-FITC, according to manufacturer´s instructions. Since Arg1 and iNOS are intracellular proteins, the cells were fixed and permeabilized beforehand with the BD Cytofix/Cytoperm™ Kit for 30 min at 4 °C. These studies were performed with macrophages from four independent blood donors, each with one technical replicate.

### Cytokine analysis

Macrophages were stimulated with BrM-TC SNs for 48 h, after which BrM-TC SNs were removed. The cells were then allowed to release cytokines in standard cell culture medium for another 24 h, at a density of 1x10^6^ cells/mL. The levels of IL-6, IL-8, IL-10 and VEGF were determined using DuoSet^®^ ELISA kits, according to the manufacturer´s recommendations. The OD values were measured at 450/540 nm on a TECAN plate reader. These studies were performed with macrophages from five independent blood donors, each with two technical replicates.

### T-cell isolation, CFSE labeling and stimulation

Peripheral blood from healthy donors was diluted 1:1 with PBS and subjected to density gradient centrifugation using Pancoll. The T-cells were isolated from the mononuclear cell fraction using the Pan T Cell Isolation Kit, according to manufacturer´s instructions. Isolated T-cells were then labelled with CFSE Cell Division Tracker Kit, according to manufacturer´s instructions. CFSE-labelled T-cells were then incubated in regular cell culture medium supplemented with plate-bound 5 µg/ml CD3 and 5 µg/ml CD28 for 24 h at 37 °C. Prior to the co-culture experiments, the pre-stimulated macrophages were washed with PBS to eliminate BrM-TC supernatant carryover. Macrophages and T-cells were co-cultured at a ratio of 1:2 for 72 h at 37 °C. T-cell viability was assessed by Propidium Iodide staining and flow cytometry to define the T-cell gating strategy of viable T-cells. These studies were performed with T-cells from three independent blood donors, each with once technical replicate.

### Statistical analysis

Statistical analysis was performed using a paired Student’s t-test to evaluate the effect of each individual patient-derived BrM-TC sample independently against its own matched internal control (unconditioned, regular cell culture medium) within the same experimental run. No pooling of different patient isolates into categorical groups was performed. For assays involving immune cells, an independent experiment was defined as using cells derived from a distinct healthy blood donor; for HUVEC assays, it corresponded to biological replicates using HUVEC at different passages. To comprehensively display data uncertainty and variance beyond binary significance thresholds, all figures present the Mean and SD, and all significant p-values are exactly reported.

## Results

### Modulation of endothelial cells by BrM-TCs of different origins

**Figure 1:**
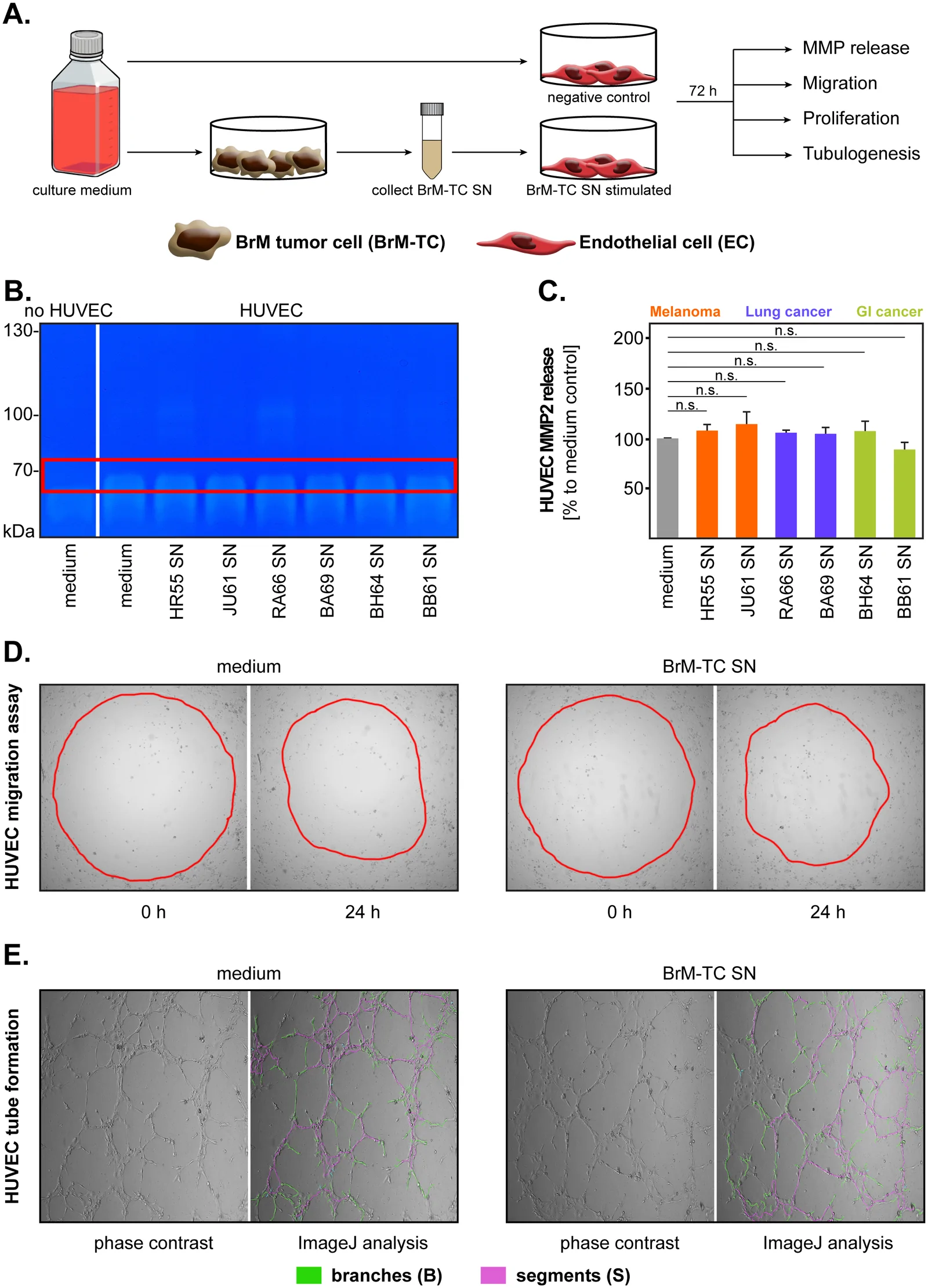
Effect of BrM-TC SNs on EC-induced matrix degradation, migration and tubulogenesis. **(A)** Schematic representation of EC stimulation with BrM-TC SNs and subsequent analysis. **(B)** Representative gelatin zymography of HUVEC stimulated with BrM-TC SNs versus medium control. Cell culture medium without HUVEC (first lane) was used to determine the basal MMP2 levels in these samples. HUVEC-derived MMP2 is indicated by a red frame. **(C)** BrM-TC SNs did not significantly affect the release of MMP2 by HUVEC. Shown are the means + S.D. of three independent experiments/biological replicates. Statistical analysis was performed with the paired t-test. **(D)** Representative micrographs of HUVEC migration upon stimulation with BrM-TC SNs (right panels) versus medium control (left panels). The red lines mark the closure of the “gap” at 0 h (pre-migration status) and 24 h (post-migration status). **(E)** ImageJ analysis of the tube formation assays in HUVEC stimulated with BrM-TC SNs (right panels) versus medium control (left panels). The branches are shown in green and the segments in magenta.

To investigate the role of BrM-TCs on angiogenesis, we prepared conditioned supernatants (SNs) from tumor cells isolated from melanoma, lung and GI BrMs, and subsequently used them to stimulate the ECs for 72 h. We then examined several critical steps of the angiogenic cascade, including the degradation of the basal membrane (as determined by the release of MMPs by ECs), as well as the EC migration, proliferation and tubulogenesis. ECs stimulated with regular cell culture medium served as control in all assays (**Figure 1A**). For this set of studies, we used HUVEC as an EC model. The release of MMPs by HUVEC was assessed at 72 h post-stimulation by gelatin zymography. The results showed that HUVEC released MMP2, but not MMP9 (**Figure 1B**). However, no significant difference in MMP2 release was observed between HUVEC stimulated with BrM-TC SNs and those from the control group (**Figure 1C**). The effect of BrM-TC SNs on EC migration was determined using the Oris™ system, by quantifying the “gap closure”, as indicated by the ImageJ analysis of the cell-free area at 0 h (pre-migration status) vs 24 h (post-migration status) (**Figure 1D**). BrM-TC SNs showed no significant effect on HUVEC migration compared to medium control (**Supplementary Figure 1A**). To assess EC proliferation, we performed MTT assays at 4 days post-stimulation, but found no significant difference in the metabolic activity of HUVEC stimulated with BrM-TC SNs compared to medium control (**Supplementary Figure 1B**). To evaluate EC tubulogenesis, we performed tube formation assays, and quantified the development of branches and segments using the ImageJ software at 5 h post-stimulation (**Figure 1E**). Also in this case, we observed no significant effect either on the number (**Supplementary Figure 1C**) or on the length (**Supplementary Figure 1D**) of branches and segments in HUVEC stimulated with BrM-TC SNs compared to medium control. Overall, these findings suggest that BrM-TCs do not exert a direct effect on the basal membrane degradation by HUVECs, nor do they directly influence HUVEC migration, proliferation or tubulogenesis under the tested conditions.

### Modulation of TAM phenotype by BrM-TCs of different origins

**Figure 2:**
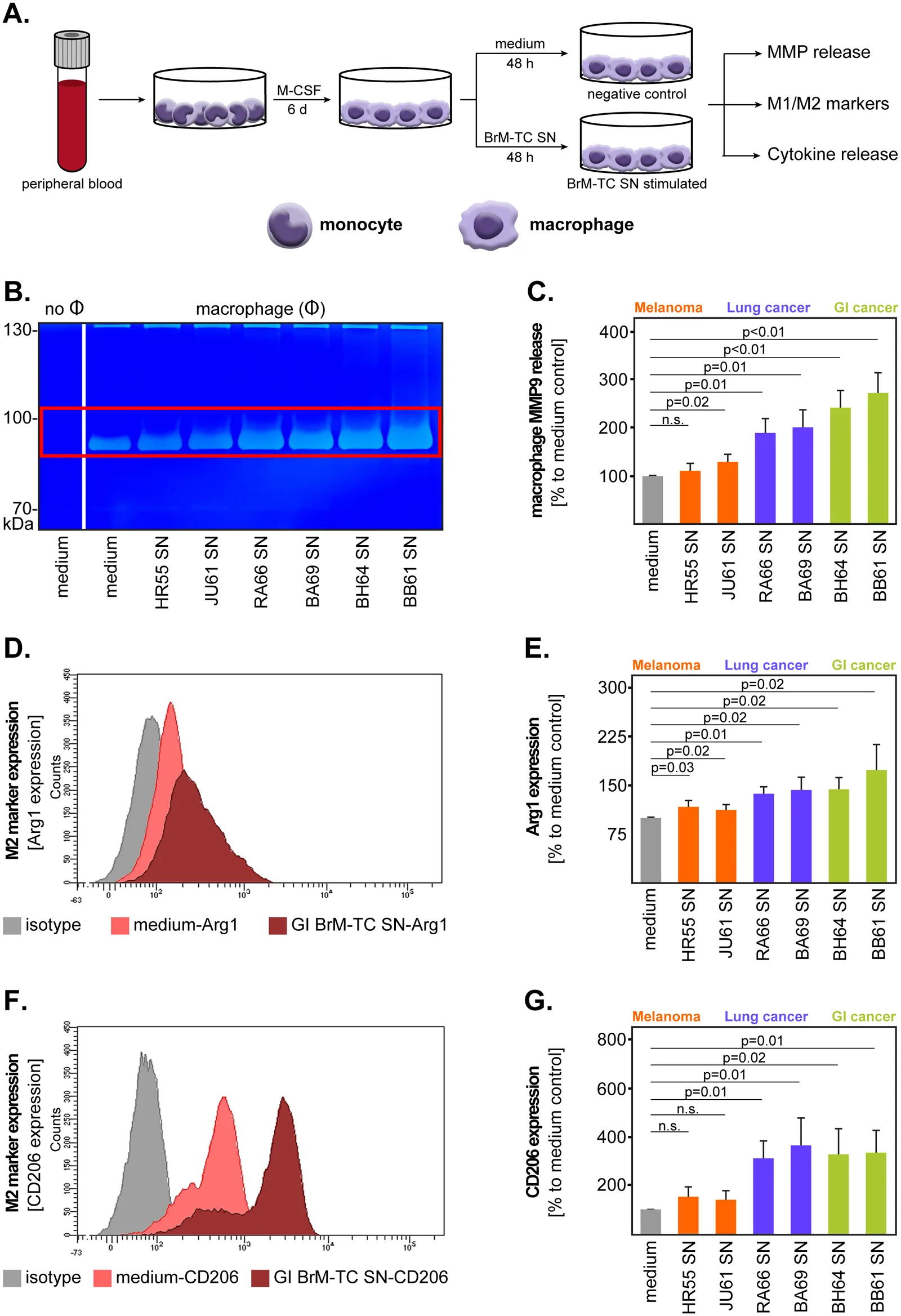
Effect of BrM-TC SNs on macrophage-derived MMP release and M1/M2 marker expression. **(A)** Schematic representation of monocyte isolation, differentiation, stimulation conditions, and read-outs. **(B)** Representative gelatin zymography of macrophages stimulated with BrM-TC SNs versus medium control. Cell culture medium without macrophages was used to determine the basal MMP9 levels in these samples. Macrophage-derived MMP9 is indicated by a red frame. **(C)** Macrophages stimulated with lung and GI BrM-TC SNs released significantly more MMP9 than those stimulated with regular cell culture medium, whereas melanoma BrM-TC SNs induced only a minor increase. Representative flow cytometric analysis of M2 markers **(D)** Arg1 and **(F)** CD206 in macrophages stimulated with GI BrM-TC SNs or medium control. Lung and GI BrM-TC SNs significantly increased the expression of **(E)** Arg1 and **(G)** CD206 compared to medium control, while melanoma BrM-TC SNs had a much weaker effect. Shown are the means + S.D. of macrophages from four independent blood donors. All statistical analyses were performed with the paired t-test.

To elucidate the effect of BrM-TCs on TAM polarization, we isolated peripheral blood monocytes and cultured them in regular cell culture medium supplemented with M-CSF for six days to induce their differentiation into macrophages. After removing the M-CSF supplemented medium, macrophages were stimulated with the same BrM-TC SNs as above or with medium control for 48 h. Subsequently, the SNs were removed and the macrophages were incubated in fresh cell culture medium for another 24 h, followed by the analysis of MMP release, M1/M2 marker expression, and release of pro-angiogenic and pro-inflammatory cytokines (**Figure 2A**). The release of MMPs by macrophages was analyzed by gelatin zymography. The results showed that macrophages released MMP9, but not MMP2 (**Figure 2B**).

Interestingly lung and GI BrM-TC SNs induced macrophages to release significantly more MMP9 compared to medium control, while melanoma BrM-TC SNs had a much weaker effect (**Figure 2C**).

To characterize in more detail the macrophage polarization state, we analyzed the expression of several markers commonly associated with the M1 phenotype (iNOS and CD86) or the M2 phenotype (Arg1, CD163 and CD206) of the TAMs. The results showed that macrophages stimulated with lung and GI BrM-TC SNs had a significantly higher Arg1 expression compared to medium control. Although melanoma BrM-TC SNs also increased the Arg1 levels, their effect was less pronounced (**Figures 2D, E**). Similarly, macrophages stimulated with lung and GI BrM-TC SNs had a significantly higher CD206 expression compared to medium control, whereas melanoma BrM-TC SNs showed a similar effect to medium control (**Figures 2F, G**). Both GI BrM-TC SNs and one of the lung BrM-TC SN significantly upregulated the expression of CD163 in macrophages compared to the medium control. In contrast, melanoma BrM-TC SNs did not have a significant effect on the CD163 expression (**Supplementary Figure 2A**). Regarding expression of M1 markers, the GI BrM-TC SNs significantly reduced iNOS expression of macrophages compared to medium control. The lung BrM-TC SNs did not significantly reduce iNOS expression in macrophages, while one of the melanoma BrM-TC SN even increased it (**Supplementary Figure 2B**). None of the BrM-TC SN had a significant effect on the expression pattern or levels of CD86 (**Supplementary Figure 2C**).

**Figure 3:**
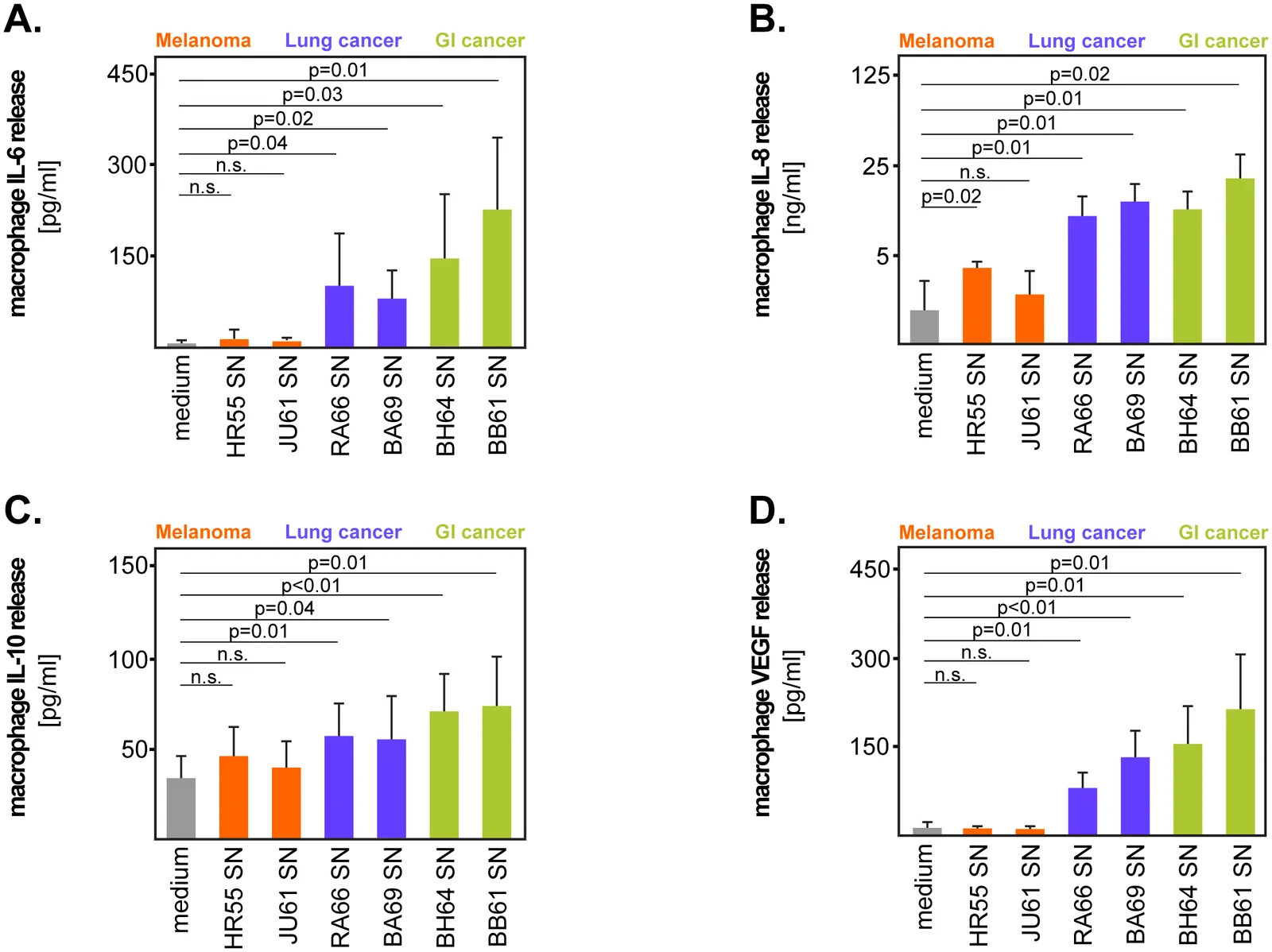
Effect of BrM-TC SNs on macrophage-derived cytokine release. Lung and GI BrM-TC SNs significantly promoted the release of M2-associated cytokines by macrophages: IL-6 **(A)**, IL-8 **(B)**, IL-10 **(C)**, and VEGF **(D)**, compared to medium control. In contrast, melanoma-derived SNs induced only modest effects, with cytokine levels largely comparable to those of the medium control. Shown are the means + S.D. of macrophages from five independent blood donors. All statistical analyses were performed with the paired t-test.

To further define these TAM polarization states, we determined the release of several pro-inflammatory and pro-angiogenic factors, such as IL-6, IL-8, IL-10 and VEGF using ELISA. The results showed that macrophages stimulated with lung and GI BrM-TC SNs released significantly higher levels of IL-6 (**Figure 3A**), IL-8 (**Figure 3B**), IL-10 (**Figure 3C**) and VEGF (**Figure 3D**) compared to medium control. While one melanoma BrM-TC SN also induced a significant IL-8 release (**Figure 3B**), the levels of released cytokines were generally much lower in the macrophages stimulated with melanoma BrM-TC SNs compared to those stimulated with lung and GI BrM-TC SNs. Taken together, these results indicate that lung and GI cancer, but not melanoma BrM-TCs, polarize macrophages toward an M2-like TAM phenotype.

### Modulation of T-cell proliferation by TAMs in BrM of different origins

**Figure 4:**
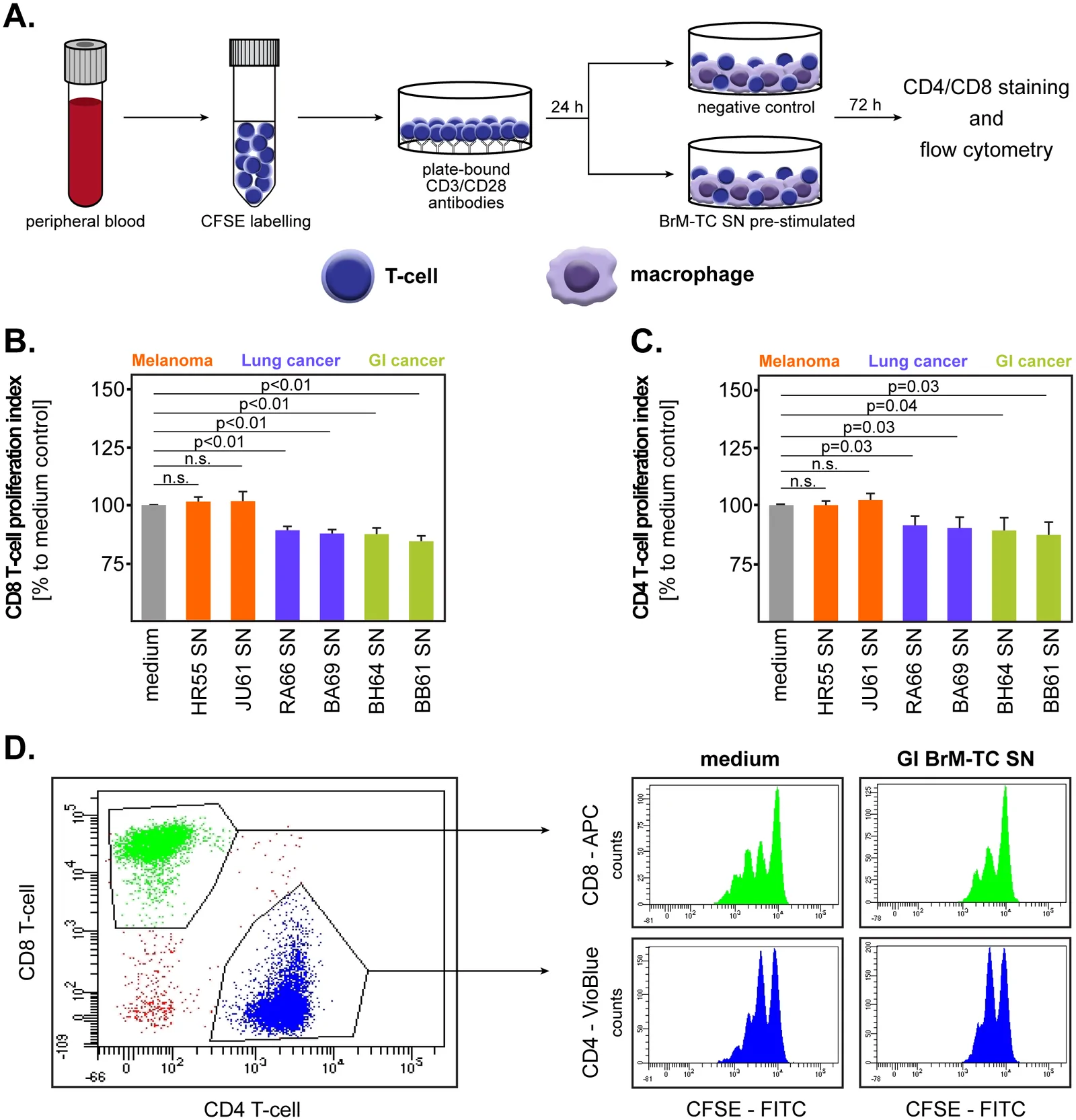
Effect of macrophages pre-stimulated with BrM-TC SNs on T-cell proliferation. **(A)** Schematic representation of T-cell isolation, CFSE labelling, stimulation, co-culture with macrophages, and analysis. Macrophages stimulated with lung and GI cancer BrM-TC SNs significantly reduced the proliferation index of **(B)** CD8 T-cells and **(C)** CD4 T-cells compared to medium control, while melanoma-derived SNs showed an effect similar to the control. Shown are the means + S.D. of T-cells from three independent blood donors. All statistical analyses were performed with the paired t-test. **(D)** Representative flow cytometric analysis showing the gating strategy and the CFSE signal dilution for CD8 (green) and CD4 T-cells (blue).

Finally, we sought to explore the functional relevance of the observed differences in TAM polarization by BrM-TC SNs of different origins, and examined their effect on T-cells proliferation. To this end, T-cells were isolated from peripheral blood, labelled with CFSE, and activated with plate-bound CD3/CD28 for 24 h to induce proliferation. The activated T-cells were then co-cultured for 72 h in fresh culture medium with macrophages that had been isolated and polarized as described above (see **Figure 2A**), using a macrophage to T-cell ratio of 1:2. Importantly, to prevent a direct effect of the BrM-TC SNs on the T-cells, the pre-stimulated macrophages were washed to remove the BrM-TC SNs before being co-cultured with the T-cells. Furthermore, both macrophages and T-cells were isolated from the same blood donor. Following co-incubation, T-cells were stained for CD4/CD8, and the CFSE signal in both subsets of T-cells was evaluated by flow cytometry (**Figure 4A**). The results showed that macrophages pre-stimulated with lung and GI cancer BrM-TC SNs significantly suppressed the proliferation of both CD8 and CD4 T-cells, as indicated by their reduced proliferation index compared to medium control. In contrast, macrophages pre-stimulated with melanoma BrM-TC SNs had similar effects to the medium control (**Figures 4B, C**). The gating strategy and the flow cytometric analysis of the CFSE staining in CD8 and CD4 T-cells is shown in **Figure 4D**. Similarly, macrophages pre-stimulated with lung and GI cancer but not melanoma BrM-TC SNs significantly reduced the division, expansion, and replication indices of both CD8 (**Supplementary Figures 3A, C, E**) and CD4 T-cells (**Supplementary Figures 3B, D, F**). Taken together, these results suggest that lung and GI cancer, but not melanoma BrM-TCs, induce an immunosuppressive TAM phenotype in the BrM microenvironment.

## Discussion

Tumor cells (TCs) modulate cancer progression through immune regulation and angiogenesis (reviewed in (36–38)). Although TAMs are established regulators of tumor progression (for recent reviews see (39–41)), their role in BrM remains poorly understood, with current research largely limited to OMICS-based studies in lung and breast cancer BrMs. Here, we demonstrate that TCs isolated from lung and GI BrM induced an M2-like phenotype in TAMs, characterized by high levels of released MMP9, IL-6, IL-8, IL-10 and VEGF alongside an elevated expression of Arg1 and CD206. In contrast, melanoma BrM-TCs did not induce this M2-like polarization. Furthermore, TAMs polarized by lung and GI, but not melanoma BrMs, suppressed CD8 and CD4 T-cell proliferation, underscoring the functional implications of TAM heterogeneity across BrMs of different origins.

While this pattern points toward a potential tissue-of-origin-dependent effect, our study is limited by three methodological considerations. First, the small number of biological replicates (n = 2 patient BrM-TC isolates per group) restricts our ability to generalize these observations to the broader patient population. Because the individual patient serves as our unit of inference, patient-specific or isolate-specific variations cannot be excluded and may influence the observed trends. Therefore, these observations must be interpreted with caution and are strictly exploratory and hypothesis-generating. Larger, independent patient cohorts are imperative to validate whether these trends represent true categorical differences between lung/GI and melanoma BrMs. Second, while the experimental design used in this study provides a practical, widely accepted *ex vivo* model-specific polarization assay for human monocyte-derived macrophages, it does not fully recapitulate the physiological complexity of the macrophage populations in BrMs or the cellular interaction within the BrM microenvironment. Critically, our *in vitro* approach utilizing tumor-conditioned medium only captures paracrine, soluble signaling and completely lacks direct physical cell-cell contact between tumor cells and macrophages, which heavily influences immune responses *in vivo*. Furthermore, this simplified model isolates macrophage-tumor cell crosstalk by excluding other vital components of the brain microenvironment, such as reactive astrocytes, resident microglia, and extracellular matrix remodeling, all of which deeply modulate macrophage polarization and function. Additionally, the use of M-CSF for the initial 6-day differentiation of monocytes may also bias the macrophages toward a more permissive baseline phenotype. Therefore, our findings should be interpreted within the context of this experimental system and its inherent limitations. Third, given the well-recognized limitations of the rigid M1/M2 dichotomy, the term “M2-like” is strictly employed as an operational descriptor for the specific immunosuppressive and pro-tumorigenic phenotypic shift observed in our model, rather than as a definition of a terminally differentiated, static macrophage subset. Although our study does provide a comprehensive initial panel of macrophage markers, the inclusion of additional targets such as PD-L1, CXCL10, or TREM2 remains an important avenue for future validation.

The observed variations in TAM modulation across BrMs align with recent studies demonstrating compositional heterogeneity based on primary tumor sites. Xing et al. identified a hypoxia- and vasculature-associated M2-like immunosuppressive TAM subset (MO4), enriched in lung and GI BrMs compared to melanoma BrMs (42). Combining RNAseq, protein arrays and spatial tissue analysis, Klemm et al. elegantly showed that lung BrMs were enriched in TAMs compared to melanoma BrMs. While these macrophages lacked a classical M1-M2 polarization pattern, they displayed a highly immunosuppressive and pro-angiogen phenotype characterized by high expression of SPP1, INHBA and VEGFA (43). Similarly, Berghoff et al. demonstrated that melanoma BrMs harbored significantly less activated microglia/macrophages compared to lung BrMs (44). This is further supported by Ratzabi et al., who showed in murine models that melanoma BrMs were significantly less infiltrated with CD206-positive TAMs compared to lung BrMs (26). In contrast to the above, Gonzalez et al. reported higher TAM frequencies in melanoma BrMs compared to lung and GI BrMs. They further defined two major TAM subsets with M2-like features and an anti-tumor phenotype, respectively that were equally present in all BrM types. Notably, this study included only one GI BrM, and suggested that prior patient therapies could have affected the observed immune clusters and frequencies (45).

Several additional groups defined TAM states in BrMs that closely aligned with the marker profiles reported here. Immune profiling and sequencing of paired primary and BrM lung tumors, revealed that BrM tissues were enriched in Arg1-positive M2-like TAMs and were less infiltrated by T-cells, indicative of an immunosuppressive microenvironment (46). Similarly, Kim et al. reported that lung BrMs were enriched in TAMs with high expression of the M2 marker CD163, which modulated the surrounding T-cells via both activating and inhibitory signals (47). High-dimensional single-cell profiling studies identified a unique subset of TAMs in melanoma and lung cancer BrM tissues, characterized by high expression of CD206, CD163, CD209, and PD-L1; however collective sample analysis precluded a direct comparison between these primary sites (48). In a murine model of lung BrM, Schulz et al. confirmed that TAMs strongly upregulated Arg1, CD163, IL-10, and VEGFA (8). In contrast to this prevailing immuno-suppressive view of TAMs in BrMs, Song et al. reported predominantly an M1 phenotype (49), while Kennedy et al. found an increased M2-to-M1 macrophage ratio in melanoma BrM (50). Collectively, these findings highlight substantial differences regarding the immune composition across BrMs of different origins, and underscore the functional diversity of TAMs within the BrM microenvironment.

While our functional characterization aligns with this diversity by demonstrating that BrM-TCs from different tissue origins differentially influence macrophage polarization, the exact downstream molecular mechanisms driving these distinct phenotypes remain to be fully elucidated. Since a comprehensive analysis of the BrM-TC secretome was beyond the scope of this study, we can only speculate on the potential mediators based on general TAM biology. In the BrM microenvironment, the polarization toward an M2-like, pro-tumorigenic phenotype was shown to be driven by tumor-derived soluble factors such as IL-6, IL-10, TGF-β, HIF-1α, LT-β, or specific chemokines, which subsequently activate the STAT3/6 and NF-κB signaling pathways in myeloid cells [reviewed in (14, 51, 52)]. To our knowledge, comparative secretome profiles specifically contrasting melanoma versus lung and GI BrMs remain absent from the current literature. Consequently, future studies will need to determine the exact BrM-TC derived factors that induce a differential polarization state of macrophages in these particular BrM types.

We additionally investigated the role of BrM-TCs on angiogenesis, given that TCs are established regulators of angiogenic activity (reviewed in (53–55). Surprisingly, we observed no direct effect of any of our BrM-TCs on the basal membrane degradation (MMP release by ECs), EC migration, proliferation, or tubulogenesis in this specific *in vitro* setting, despite literature implicating TCs in BrM angiogenesis (reviewed in (5, 6)). This lack of effect may partially be explained by the complex, multifactorial regulation of angiogenesis by a dynamic balance of competing pro- and anti-angiogenic factors (56). However, it must be emphasized that our EC experiments were performed strictly using HUVECs. While HUVECs are a standard model for general angiogenesis, they do not possess the unique phenotypic, transcriptomic, or barrier characteristics of brain-specific microvascular endothelial cells, nor do they recapitulate the blood-brain barrier. Additionally, evaluating the biology and functions of HUVECs after the removal of the BrM-TC supernatants might attenuate immediate paracrine dynamics. Consequently, our findings are restricted to this particular experimental model and these specific conditions, and cannot be generalized to brain-specific angiogenesis. Therefore, further studies using human brain microvascular endothelial cells (hBMECs) or more physiologically relevant blood-brain-barrier relevant models are absolutely necessary to definitively clarify the role of BrM-TCs in the cerebral angiogenesis.

In conclusion, our study provides a preliminary focused *ex vivo* functional analysis using patient-derived BrM-TC supernatants to evaluate macrophage responses across different BrM tumor origins. Given the small sample size, our exploratory data suggest that lung and GI, but not melanoma BrM-TCs, may preferentially polarize human macrophages *ex vivo* toward a tumor-promoting, M2-like phenotype, as indicated by increased MMP9 release, elevated Arg1 and CD206 expression, as well as enhanced IL-6, IL-8, IL-10, and VEGF secretion under these specific experimental conditions. These functional hypothesis-generating comparisons complement existing descriptive spatial and single-cell datasets regarding inter-origin heterogeneity in BrMs, but require extensive validation in larger cohorts before definitive conclusions can be drawn. While our observations regarding a lack of direct angiogenic effects are limited to the HUVEC model and require further characterization in brain-specific systems, our results nevertheless highlight the heterogeneity of BrMs, and emphasize the need for more detailed and differentiated studies to better understand the pathophysiology of these tumors.

## Data Availability

The original contributions presented in the study are included in the article/**Supplementary Material**. Further inquiries can be directed to the corresponding authors.
