# Supplementary material for: The paracrine effects of tumor cells on endothelial cells and macrophages in brain metastases with different primary sites
Source: Front Immunol. 2026 Aug 26;17:1872801. doi: 10.3389/fimmu.2026.1872801 (PMC13553479; doi:10.3389/fimmu.2026.1872801)
Supplement: Supplementary file 1 [file DataSheet1.pdf]

Supplementary Table S1. List of materials used in the study according to category.

		Manufacturer	Product No.
Antibodies	APC anti-human Arginase 1 Antibody	Biolegend, San Diego, CA, USA	369705
	APC Mouse IgG2b, κ Isotype Ctrl Antibody	Biolegend, San Diego, CA, USA	400322
	CoraLite® Plus 488-conjugated iNOS Polyclonal Antibody	Proteintech, Rosemont, IL, USA	CL488-18985
	CoraLite® Plus 488 rabbit IgG Isotype Control Recombinant Antibody	Proteintech, Rosemont, IL, USA	CL488-98136
	CD86 Antibody, anti-human, APC-Vio® 770, REAfinity™	Miltenyi Biotech, Bergisch Gladbach, Germany	130-116-163
	CD163 Antibody, anti-human, PE, REAfinity™	Miltenyi Biotech, Bergisch Gladbach, Germany	130-112-286
	CD206 Antibody, anti-human, VioBlue®	Miltenyi Biotech, Bergisch Gladbach, Germany	130-127-809
	HumanKine® recombinant human M-CSF protein	Proteintech, Rosemont, IL, USA	HZ-1192
	REA Control Antibody, human IgG1, APC-Vio 770, REAfinity	Miltenyi Biotech, Bergisch Gladbach, Germany	130-113-447
	REA Control Antibody, human, PE	Miltenyi Biotech, Bergisch Gladbach, Germany	130-113-450
	REA Control Antibody, human igG1, VioBlue, REAfinity	Miltenyi Biotech, Bergisch Gladbach, Germany	130-113-454
	Ultra-LEAF™ Purified anti-human CD3 Antibody	Biolegend, San Diego, CA, USA	300438
	Ultra-LEAF™ Purified anti-human CD28 (Superagonistic) Antibody	Biolegend, San Diego, CA, USA	377804
Kits	BD Cytofix/Cytoperm™ Fixation/Permeabilization Kit	BD Biosciences, Heidelberg, Germany	554714
	CFSE Cell Division Tracker Kit (DMSO)	Biolegend, San Diego, CA, USA	423801
	Human VEGF DuoSet ELISA	RnD Systems, Minneapolis, MN, USA	DY293B-05
	Human IL-6 DuoSet ELISA	RnD Systems, Minneapolis, MN, USA	Dy206-05
	Human IL-8 DuoSet ELISA	RnD Systems, Minneapolis, MN, USA	DY208-05
	Human IL-10 DuoSet ELISA	RnD Systems, Minneapolis, MN, USA	DY217B-05
	Oris™ 3D Embedded Invasion Assay Kit	Platypus Technologies, Madison, WI, USA	EIA1

	Pan Monocyte Isolation Kit, human	Miltenyi Biotech, Bergisch Gladbach, Germany	130-096-537
	Pan T Cell Isolation Kit, human	Miltenyi Biotech, Bergisch Gladbach, Germany	130-096-535
**Chemicals and disposable material**	Acetic acid	Carl Roth, Karlsruhe, Germany	3738.2
	Ammonium peroxydisulphate	Carl Roth, Karlsruhe, Germany	9592.3
	Aqua B. Braun	B. Braun SE, Melsungen, Germany	0082479E
	Brilliant blue G 250	Carl Roth, Karlsruhe, Germany	9598.1
	Bromophenol blue	Carl Roth, Karlsruhe, Germany	A512.2
	Cultrex Reduced Growth Factor Basement Membrane Extract	Bio-Techne, Minneapolis, MN, USA	3433-005-01
	FBS Supreme	Pan Biotech, Aidenbach, Germany	P30-3031
	Gelatine 180 bloom	Carl Roth, Karlsruhe, Germany	4274.4
	Glycerin	Carl Roth, Karlsruhe, Germany	3783.1
	Hydrochloric acid fuming 37%	Carl Roth, Karlsruhe, Germany	X942.1
	Isopropanol (2-propanol)	Morphisto, Frankfurt, Germany	19288.00250
	Macrophage Detachment Solution	PromoCell, Heidelberg, Germany	C-41330
	Methanol	Carl Roth, Karlsruhe, Germany	0082.2
		Carl Roth, Karlsruhe, Germany	T145.2
	Oris Collagen I (rat Tail)	Platypus Technologies, Madison, WI, USA	24B2801
	Pageruler™ Plus prestained Protein Ladder	Thermo Fisher Scientific, Waltham, MA, USA	26619
	Pancoll human	Pan Biotech, Aidenbach, Germany	P04-60500
	Penicillin-Streptomycin	Thermo Fisher Scientific, Waltham, MA, USA	15140122
	ROTI®Cell 10x PBS	Carl Roth, Karlsruhe, Germany	9150.1
	Roti®Cell DMEM high glucose	Carl Roth, Karlsruhe, Germany	9007.1
	Rotiphorese®Gel 40 (29:1)	Carl Roth, Karlsruhe, Germany	A515.1
	SDS ultra pure	Carl Roth, Karlsruhe, Germany	2326.4
	Sodium chloride	Carl Roth, Karlsruhe, Germany	HN00.2
	TEMED	Carl Roth, Karlsruhe, Germany	2367.3
	Thiazol blue	Carl Roth, Karlsruhe, Germany	4022.1
	Tris	Carl Roth, Karlsruhe, Germany	4855.2
	Triton X-100	Carl Roth, Karlsruhe, Germany	3051.4

Software	BD FACSDiva™	BD Biosciences, Heidelberg, Germany	n.a.
	ImageJ 1.48v	NIH, Bethesda, MD, USA	n.a.
	Magellan™ 7.2	Tecan Life Sciences, Männedorf, Switzerland	n.a.
Equipment	BD FACSCanto II flow cytometer	BD Biosciences, Heidelberg, Germany	n.a.
	BZ-X810 fluorescence microscope	Keyence, Neu-Isenburg, Germany	n.a.
	TECAN plate reader	Tecan Life Sciences, Männedorf, Switzerland	n.a.

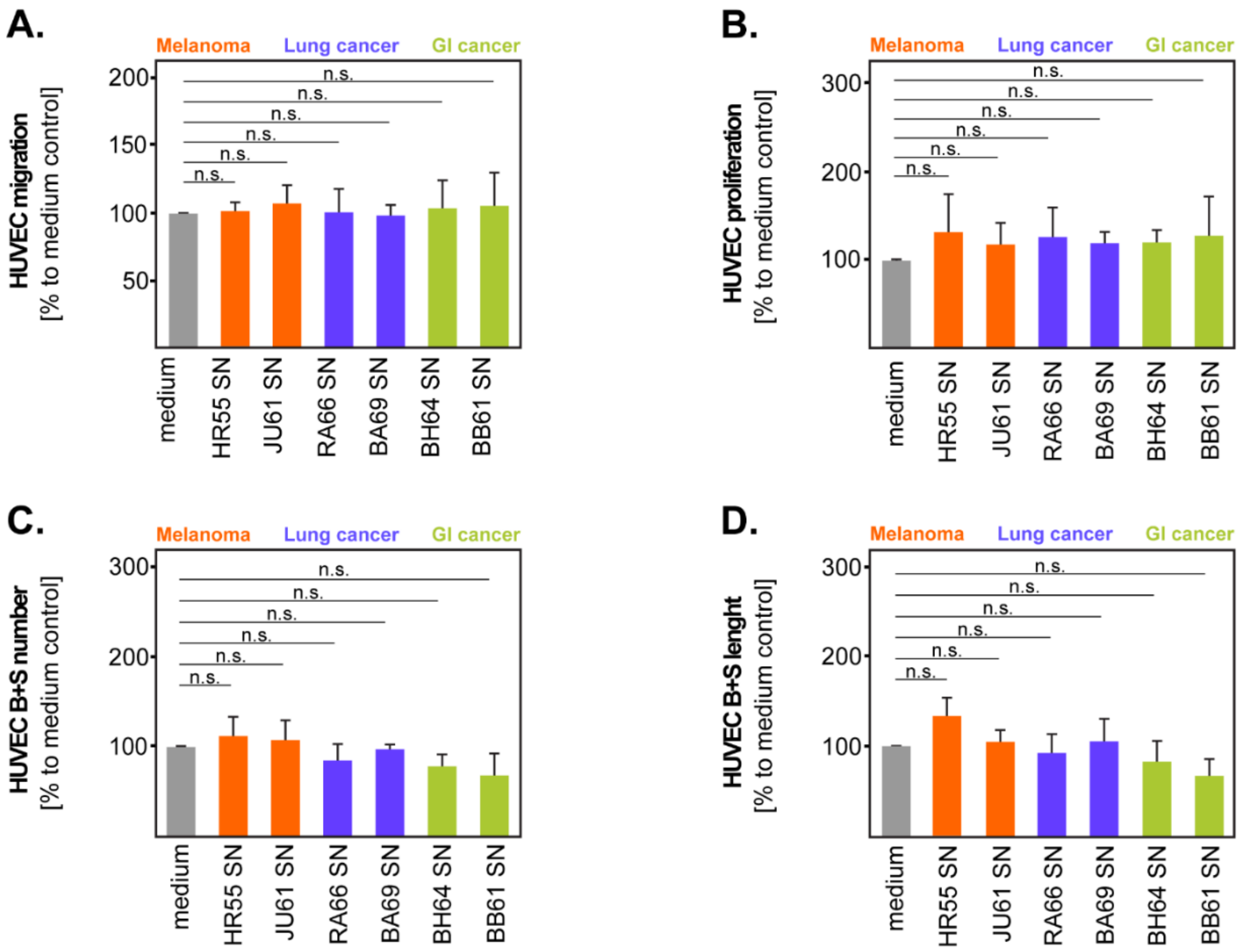

Supplementary Figure S1. Effect of BrM-TC SNs on EC migration, proliferation and tubulogenesis. (A) BrM-TC SNs did not significantly affect the migration of HUVEC cells. **(B)** HUVEC proliferation at 4 days post-stimulation, as measured by the MTT assay. BrM-TC SNs had no significant effect on the proliferation of HUVEC cells. None of the BrM-TC SNs significantly modulated the **(C)** number and **(D)** length of branches and segments in HUVEC cells compared to medium control. Shown are the means + S.D. of five **(A, B)** and three **(C, D)** independent experiments. All statistical analyses were performed with the paired t-test.

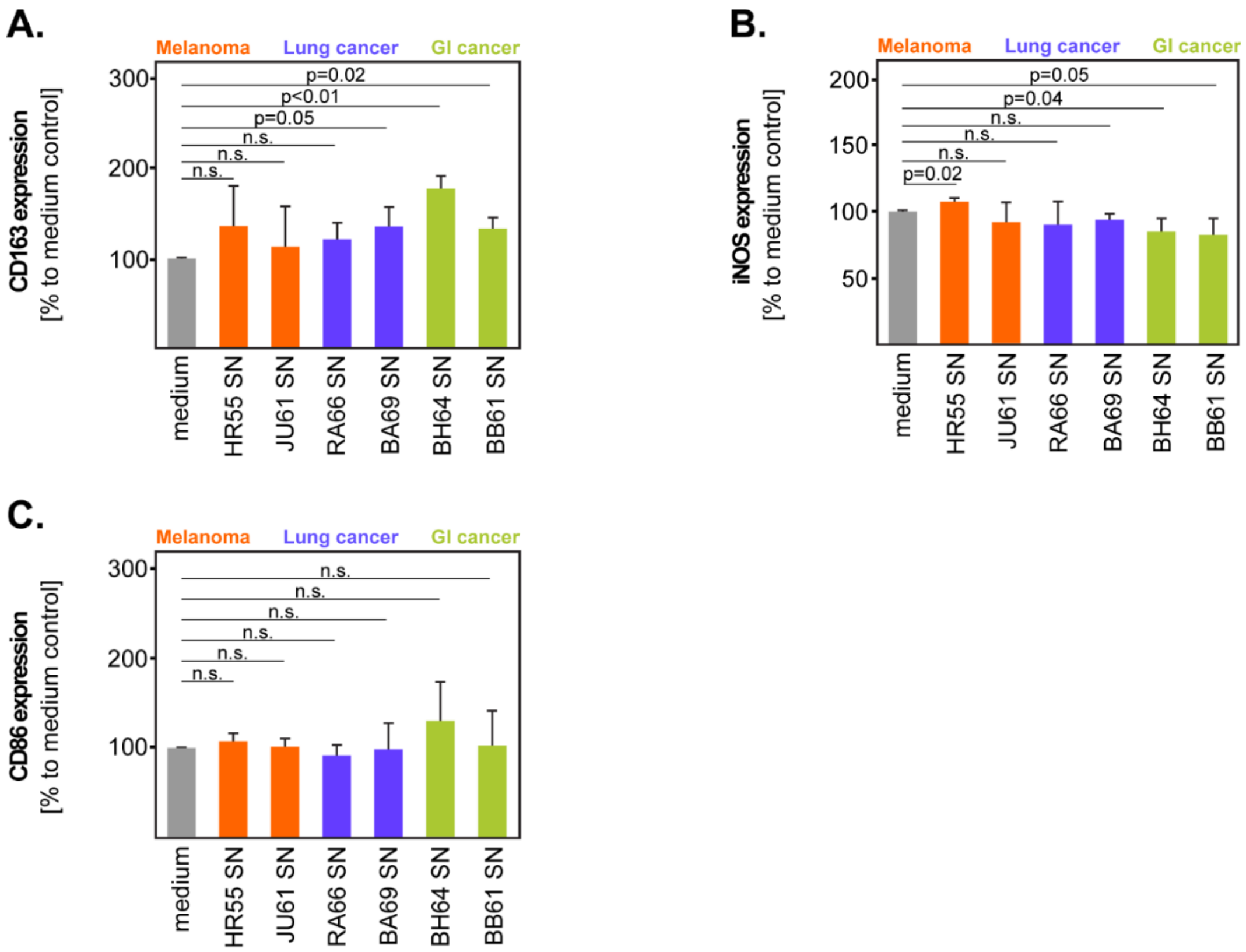

Supplementary Figure S2. Effect of BrM-TC SNs on macrophage M1/M2 marker expression. The expression levels of **(A)** CD163, **(B)** iNOS, and **(C)** CD86 in macrophages stimulated with lung, GI cancer, and melanoma BrM-TC SNs. Shown are the means + S.D. of at least three independent experiments. All statistical analyses were performed with the paired t-test.

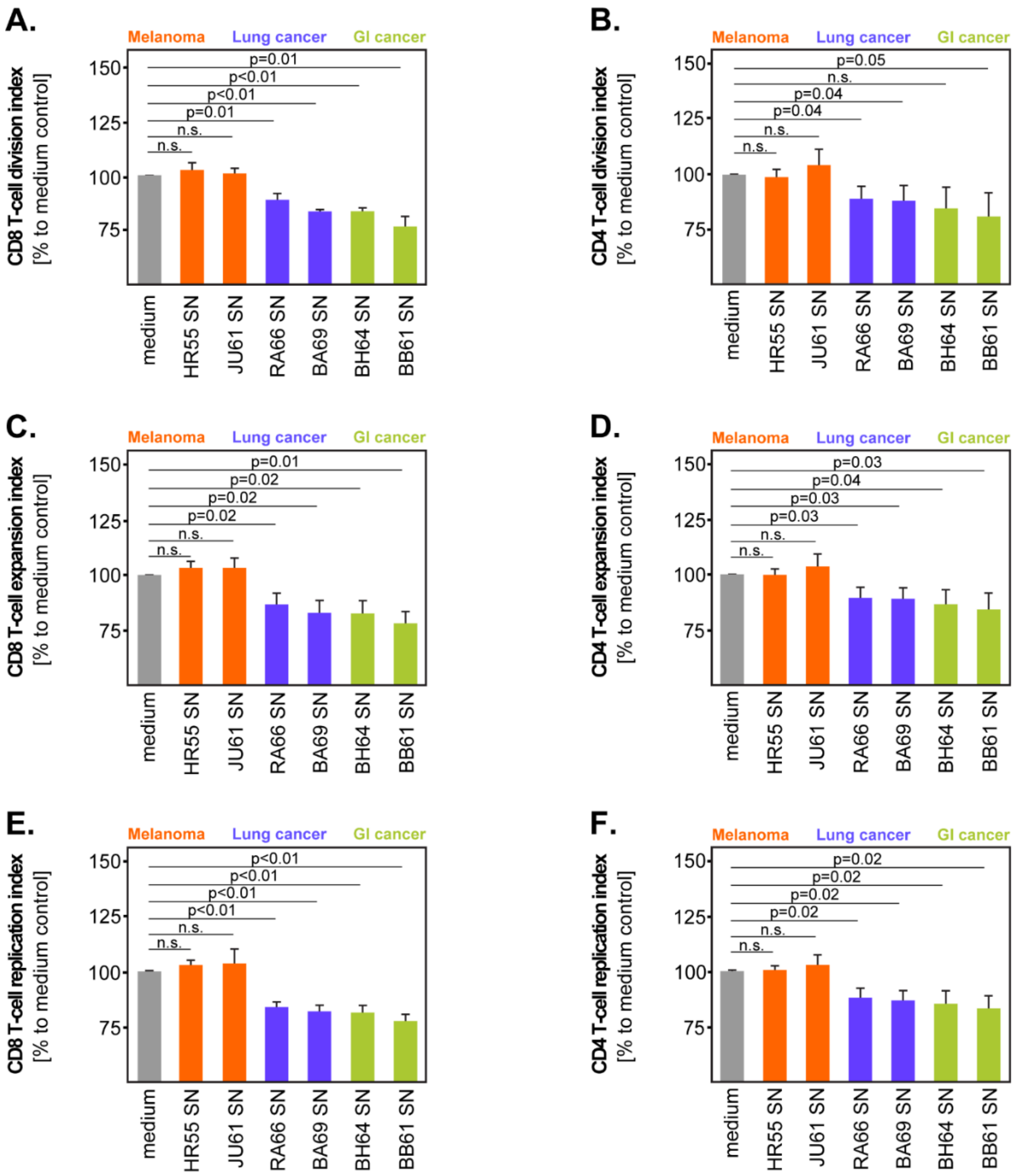

Supplementary Figure S3. Effect of macrophages pre-stimulated with BrM-TC SNs on T-cell proliferation. Macrophages stimulated with lung and GI cancer BrM-TC SNs significantly reduced the division, expansion and replication indices of CD8 **(A, C, E)** and CD4 T-cells **(B, D, F)** compared to medium control, respectively. In contrast, melanoma-derived SNs showed effects similar to the medium control group. Shown are the means + S.D. of three independent experiments. All statistical analyses were performed with the paired t-test.
